# Supplementary material for: Reconstructing B-cell receptor sequences from short-read single-cell RNA sequencing with BRAPeS
Source: Life Sci Alliance. 2019 Aug 26;2(4):e201900371. doi: 10.26508/lsa.201900371 (PMC6709718; doi:10.26508/lsa.201900371)
Supplement: Supplementary file 2 [file LSA-2019-00371_TableS2.docx]

**Table S2:** Detailed description of the number of productive CDR3 reconstructions for the original long reads and 30bp sequencing

|  | **Human (n = 174)** | | | **Mouse (n = 200)** | | |
| --- | --- | --- | --- | --- | --- | --- |
|  | **Heavy** | **Light (kappa or Lambda)** | **Both kappa and Lambda** | **Heavy** | **Light (kappa or Lambda)** | **Both kappa and Lambda** |
| BASIC - long read | 171 (98.3%) | 174 (100%) | 1 (0.6%) | 182 (91%) | 190 (95%) | 0 (0%) |
| VDJPuzzle - long read | 162 (93.1%) | 172 (98.9%) | 17 (9.77%) | 184 (92%) | 196 (98%) | 3 (1.5%) |
| BRAPeS - 30bp | 173 (99.4%) | 170 (97.7%) | 14 (8%) | 175 (87.5%) | 200 (100%) | 3 (1.5%) |
| BASIC - 30bp | 164 (94.3%) | 171 (98.3%) | 0 (0%) | 109 (54.5%) | 191 (95.5%) | 0 (0%) |
| VDJPuzzle - 30bp | 149 (85.6%) | 172 (98.9%) | 6 (3.45%) | 93 (46.5%) | 198 (99%) | 1 (0.5%) |
