# Supplementary material for: Reconstructing B-cell receptor sequences from short-read single-cell RNA sequencing with BRAPeS
Source: Life Sci Alliance. 2019 Aug 26;2(4):e201900371. doi: 10.26508/lsa.201900371 (PMC6709718; doi:10.26508/lsa.201900371)
Supplement: Supplementary file 3 [file LSA-2019-00371_TableS3.docx]

**Table S3:** Detailed description of the number of productive CDR3 reconstructions for the original long reads and 25bp sequencing

|  | **Human (n = 174)** | | | **Mouse (n = 200)** | | |
| --- | --- | --- | --- | --- | --- | --- |
|  | **Heavy** | **Light (kappa or Lambda)** | **Both kappa and Lambda** | **Heavy** | **Light (kappa or Lambda)** | **Both kappa and Lambda** |
| BASIC - long read | 171 (98.3%) | 174 (100%) | 1 (0.6%) | 182 (91%) | 190 (95%) | 0 (0%) |
| VDJPuzzle - long read | 162 (93.1%) | 172 (98.9%) | 17 (9.77%) | 184 (92%) | 196 (98%) | 3 (1.5%) |
| BRAPeS - 25bp | 172 (98.9%) | 171 (98.3%) | 8 (4.6%) | 156 (78%) | 199 (99.5%) | 4 (2%) |
| BASIC - 25bp | 137 (78.7%) | 173 (99.4%) | 0 (0%) | 67 (33.5%) | 190 (95%) | 0 (0%) |
| VDJPuzzle -25bp | 0 (0%) | 0 (0%) | 0 (0%) | 0 (0%) | 0 (0%) | 0 (0%) |
